# The Use of Text Messaging to Improve the Hospital-to-Community Transition in Acute Coronary Syndrome Patients (Txt2Prevent): Intervention Development and Pilot Randomized Controlled Trial Protocol

**DOI:** 10.2196/resprot.6968

**Published:** 2017-05-23

**Authors:** Emily S Ross, Brodie M Sakakibara, Martha H Mackay, David GT Whitehurst, Joel Singer, Mustafa Toma, Kitty K Corbett, Harriette GC Van Spall, Kimberly Rutherford, Bobby Gheorghiu, Jillianne Code, Scott A Lear

**Affiliations:** ^1^ Department of Biomedical Physiology and Kinesiology Simon Fraser University Burnaby, BC Canada; ^2^ Faculty of Health Sciences Simon Fraser University Burnaby, BC Canada; ^3^ Department of Physical Therapy University of British Columbia Vancouver, BC Canada; ^4^ School of Nursing University of British Columbia Vancouver, BC Canada; ^5^ Centre for Clinical Epidemiology and Evaluation Vancouver Coastal Health Research Institute Vancouver, BC Canada; ^6^ School of Population and Public Health University of British Columbia Vancouver, BC Canada; ^7^ Centre for Health Evaluation and Outcome Sciences University of British Columbia Vancouver, BC Canada; ^8^ Division of Cardiology Providence Health Care Vancouver, BC Canada; ^9^ School of Public Health and Health Systems University of Waterloo Waterloo, ON Canada; ^10^ Department of Health Research Methods, Evidence, and Impact McMaster University Hamilton, ON Canada; ^11^ Department of Medicine McMaster University Hamilton, ON Canada; ^12^ Department of Family Practice University of British Columbia Vancouver, BC Canada; ^13^ Canada Health Infoway Toronto, ON Canada; ^14^ Faculty of Education University of Victoria Victoria, BC Canada

**Keywords:** acute coronary syndrome, cardiovascular diseases, heart diseases, mobile health, text messaging, mobile phone, SMS

## Abstract

**Background:**

Acute coronary syndrome, including acute myocardial infarction (AMI), is one of the leading causes for hospitalization, with AMI 30-day readmission rates around 20%. Supporting patient information needs and increasing adherence to recommended self-management behaviors during transition from hospital to home has the potential to improve patient outcomes. Text messages have been effective in other interventions and may be suitable to provide support to patients during this transition period.

**Objective:**

The goal of this study is to pilot test a text messaging intervention program (Txt2Prevent) that supports acute coronary syndrome patients for 60 days postdischarge. The primary objective is to compare self-management, as measured by the Health Education Impact Questionnaire, between patients receiving only usual care versus those who receive usual care plus the Txt2Prevent intervention. The secondary objectives are to compare medication adherence, health-related quality of life, self-efficacy, health care resource use (and associated costs), all-cause and cardiovascular disease (CVD) readmission, and all-cause and CVD mortality rates between the 2 groups. The third objective is to assess acceptability of the text messaging intervention and feasibility of the study protocol.

**Methods:**

This is a randomized controlled trial with blinding of outcome assessors. The Txt2Prevent program includes automated text messages to patients about standard follow-up care, general self-management, and healthy living. The content of the text messages was informed by and developed based on interviews with patients, discharge materials, theoretical domains of behavior, and a clinical advisory group composed of patients, clinicians, and researchers. We will recruit 76 consecutive cardiac in-patients with acute coronary syndrome who are treated with either medical management or percutaneous coronary intervention from a hospital in Vancouver, Canada.

**Results:**

Assessments at baseline will include measures for demographic information, self-management, health-related quality of life, and self-efficacy. Assessments at follow-up will include medication adherence, readmissions, health care resource use, and mortality in addition to the reassessment of baseline measures. Baseline assessments are done in-person while follow-up assessments are completed through a combination of mailed packages and phone calls. Semistructured interviews with participants will also be performed to better understand participant experiences managing their condition and with the text messages.

**Conclusions:**

This study will determine preliminary efficacy, feasibility, and acceptability of the Txt2Prevent program to support acute coronary syndrome patients in the transition to home following hospital discharge. The results of this study will be used to inform a larger trial.

**Trial Registration:**

ClinicalTrials.gov NCT02336919; https://clinicaltrials.gov/ct2/show/NCT02336919 (Archived by WebCite at http://www.webcitation.org/6qMjEqo6O)

## Introduction

### Background

Cardiovascular disease (CVD) is one of the leading causes for hospitalization and death in Western countries [1,2]. Acute coronary syndrome (ACS) includes the diagnoses of acute myocardial infarction (AMI) and unstable angina. AMIs are the second most common reason for inpatient admissions in Canada (excluding giving birth), with over 60,000 cases [3] while ACS was the primary or secondary cause of over 1.1 million unique hospital admissions in the United States in 2010 [4]. Approximately 60% of these admissions were due to AMIs, making AMIs a leading cause for inpatient admissions [4,5]. The initial period following discharge is the highest risk for readmission, with 14% of AMI patients having an urgent readmission within 15 days of discharge and 20% of patients being readmitted by 30 days [6]. In the United States, Dharmarajan et al [7] found that AMI patients’ daily change in risk of readmission has declined by 95% by day 38 postdischarge. In Canada, the median days until readmission in ACS patients was 23 (interquartile range: 5 to 41 days) [8]. Readmissions are of concern because of the impact on patient quality of life [9] and the cost to the health care system, which has been estimated at $1 billion in the United States in 2013 [10,11].

Patients have several challenges during the transition period after discharge that can influence readmissions, including lack of support, potentially preventable adverse events, and patient inability to perform self-management behaviors [12,13,14]. During the transition period, patients report feeling overwhelmed, uncertain, and alone with physical or mental setbacks [12,15]. Patients may be confused about the information they received in the hospital [15] and may want more information once they are home because being informed often provides reassurance [12]. Additionally, up to 23% of patients may experience adverse events after discharge, such as adverse drug events or therapeutic error, of which half may be preventable or ameliorable [13]. Having better transitional care could help to identify or prevent these errors. Following hospital discharge, ACS patients also must become independent with new self-management responsibilities [12,16]. Self-management is the concept that people with chronic illness, such as CVD, must live with and manage their disease on a daily basis, which includes engaging in healthy behaviors to control or reduce the impact of their disease, communicating effectively with health professionals and caregivers, and managing physical and emotional challenges [17]. Meta-analyses have found chronic disease self-management programs have been associated with reduced hospital use (particularly in CVD and respiratory patients) [18], improvements in health behaviors such as aerobic exercise, cognitive symptom management, and communication with physicians [19], improvements in health outcomes such as glycemic control in diabetes patients and blood pressure reductions in hypertension patients [20,21], and increased quality of life and self-efficacy [22,20]. Self-management for ACS patients includes recommended behavioral changes (eg, smoking cessation, exercise, and adhering to a healthy diet) and taking their medications as prescribed. Despite this knowledge, research shows that many patients continue with unhealthy behaviors. In one study, 30 days after discharge, 35% of smokers continued to smoke and 29% of patients do not adhere to physical activity and diet recommendations [14]. The authors found nonadherence to smoking, diet, and exercise behavioral recommendations is associated with a 3.8-fold increased risk of myocardial infarction, stroke, or death at 6 months postdischarge [14]. Another study reported that within the first 7 days after discharge, 23% of cardiac medication prescriptions were not filled despite the association between medication adherence and reduced mortality [23]. Therefore, providing continuing support post–hospital discharge has the potential to affect several key factors of post-ACS management, including medication adherence, lifestyle changes, and adverse events, which in turn can impact patient experiences and outcomes.

Text messaging technology presents an opportunity to help support patients during the hospital-to-home transition. Mobile phone ownership has increased from 65% in 2004 to 92% in 2015 in the United States [24]. While mobile phone ownership is higher in younger demographics, 78% of adults ages 65 and older own a mobile phone [24], and 80% of mobile phone owners send or receive text messages making it one of the most commonly used features [25]. Text messages can provide information to patients in a manageable amount at the appropriate time point in their recovery. Messages can include reminders and prompts to engage in the recommended postdischarge behaviors. Messages are inexpensive to send and receive, and automated delivery systems do not require a significant amount of staff time to maintain. Additional benefits are that messages may be stored on the device to be accessed multiple times and do not require both the sender and the receiver to be available at the same time, such as with a standard phone call. Text messages also have a wide geographic reach, ensuring that patients do not have to travel to receive the information. In previous text messaging studies with patients with or at risk for CVD, there have been improvements in self-management behaviors, such as medication adherence [26], and increased leisure, physical activity, and walking [27]. Text message interventions have also contributed to improvements in cardiac risk factors including lowering LDL cholesterol and systolic blood pressure [28]. Additionally, a systematic review of text messaging studies in diabetes patients found improved scores on measures for self-management capacity [29]. The studies in a CVD population did not target the hospital-to-community transition period, so it is worth investigating the potential for text messaging to support CVD patients as they transition from hospital to home.

### Study Objectives

The aim of this pilot study is to test a 1-way text messaging intervention program (Txt2Prevent) composed of 48 messages that supports ACS patients for 60 days after their hospital discharge in a single-blinded randomized controlled trial. The objectives are as follows:

1. To compare self-management between participants receiving usual care versus participants receiving usual care plus the Txt2Prevent program as measured by the Health Education Impact Questionnaire (heiQ). The heiQ assesses proximal outcomes of self-management and patient education programs [30].
2. To compare health-related quality of life, medication adherence, self-efficacy, all-cause and cardiovascular-related hospital readmissions and mortality rates, and health care resource use (and associated costs) between participants receiving usual care versus those receiving usual care plus the Txt2Prevent program.
3. To assess the acceptability of the text messaging intervention program according to patient participants and the feasibility of the study protocol for study staff.

We hypothesize that the Txt2Prevent group will have improved self-management compared with usual care. This paper describes the research protocol for the pilot study [ClinicalTrials.gov NCT02336919] in accordance with the Consolidated Standards of Reporting Trials (CONSORT) eHealth checklist [31].

## Methods

### Overview

The Txt2Prevent project is a mixed methods, single-blinded randomized controlled trial with a parallel group design. Participants will be randomized to receive either usual care or usual care plus 1-way text messaging for the first 60 days postdischarge from the hospital (Txt2Prevent).

### Setting, Participants, and Recruitment

**Figure 1:**
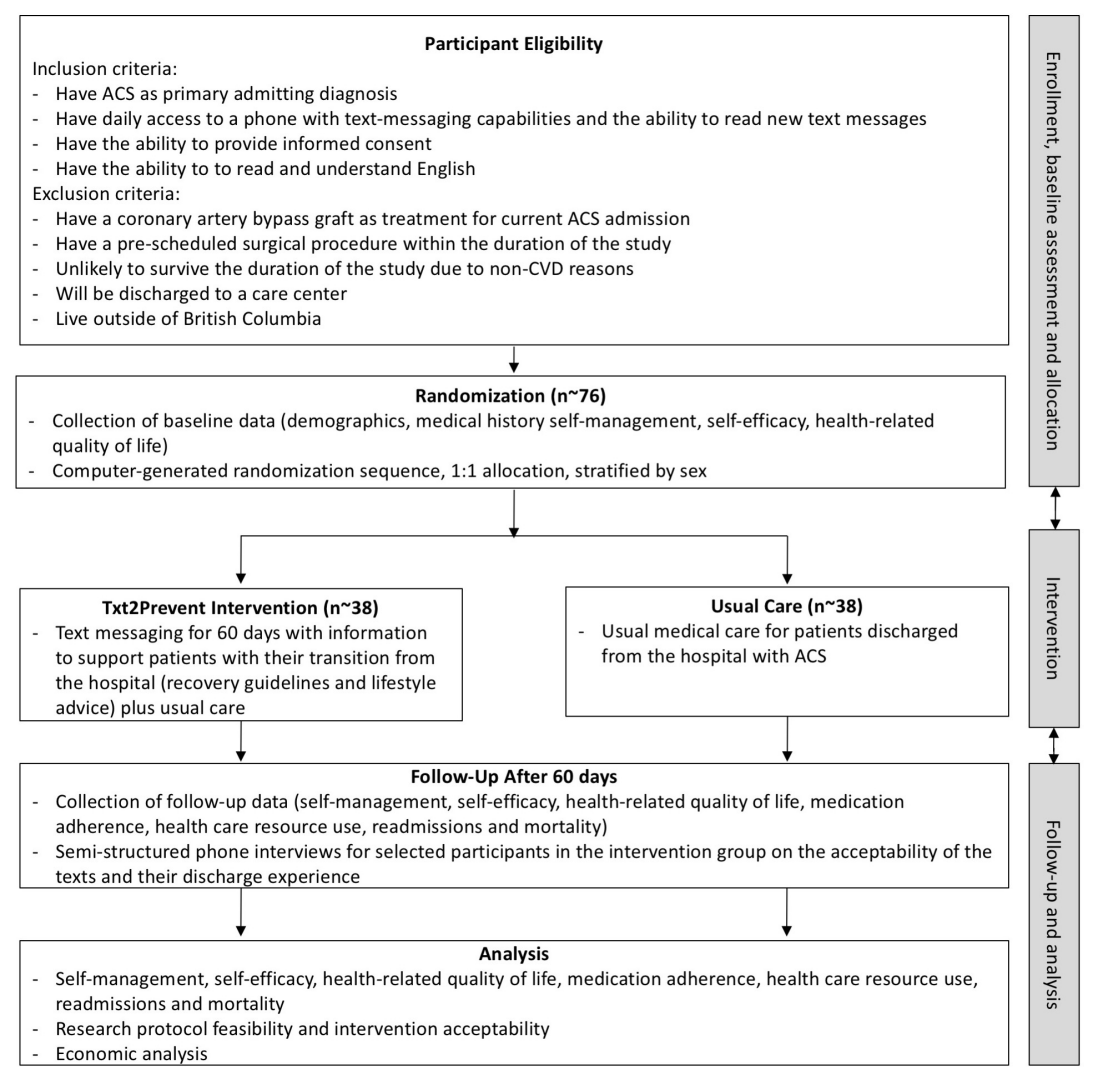
Study design and flow (ACS: acute coronary syndrome, CVD: cardiovascular disease).

Participants will be recruited from the provincial heart center located in a tertiary care hospital in Vancouver, British Columbia, Canada. This hospital serves patients from across the province, including the local metropolitan area as well as urban and rural areas. Consecutive patients admitted for ACS, as identified by clinical staff, will be screened for eligibility (see Figure 1). ACS patients whose treatment is a coronary artery bypass graft will be excluded as they have different recovery guidelines than medical management or percutaneous coronary intervention patients due to the more invasive nature of the procedure. Those who are eligible and interested will provide written, informed consent. The consent process will occur in person at the hospital. If the patient is discharged before it is possible for the research assistant to complete the written consent process, the patient may provide their phone number to study staff so they can complete an oral consent process over the phone within 7 days of discharge. The research assistant will also explain and give the participant a 1-page sheet outlining the study process and study contact information while the participant is still in the hospital or over the phone (including sending a copy through email or mail).

### Sample Size

A convenience sample of 76 participants will be enrolled in the study. This sample size is based on the feasibility of recruitment over a 6-month time period and is not determined in order to be able to detect between-group differences. Approximately 750 unique ACS patients were discharged from the target hospital during the 2012/2013 fiscal year. In a preliminary feasibility survey of patients, 14 of 28 had mobile phones with texting capabilities. Assuming 40% are eligible and 50% agree to participate [32], we expect approximately 76 patients will agree to participate over 6 months of recruiting.

### Randomization

After discharge and once the baseline assessment has been completed, participants will be randomized to receive either usual care or usual care plus the 60-day text messaging program (Txt2Prevent) using a 1:1 allocation ratio. To minimize bias, randomization will be conducted by a research assistant who is not involved in either the recruitment or data collection phases. This research assistant will use a Web-based randomization service developed through an independent research center. To ensure balance between the groups, the randomization will be stratified by sex and use block randomization with variable block sizes. The randomization research assistant will register participants for the TxT2Prevent intervention, when appropriate, and then contact all participants by phone to inform them of their group assignment. The start date for participants in the Txt2Prevent program will be documented. A letter will also be sent to all study participants’ primary care providers informing them that their patient has been enrolled in the study. Primary care providers will be identified by the participant. The primary care providers’ addresses will be obtained online from the registry maintained by the provincial licensing and regulatory body for physicians. Participants will be told to contact the research assistant who performs the randomization during the course of the study if they have any questions. Participants will be aware that the text messaging program is the intervention of interest as it is being compared to usual care.

### Study Design

#### Intervention

The intervention group will receive a total of 48 health-related text messages: 1 per day for the first 36 days, then 1 every other day for days 37 to 60 (Table 1). The text messaging program will begin the day after the participant is discharged from the hospital or, if the participant has already been discharged, immediately after the baseline questionnaires are completed. Some of the messages are time sensitive regarding their recovery (eg, the recommended timeframe to see their primary care provider after discharge), while others are general healthy living texts. Participants randomized to the intervention group will be registered through our secured, password-protected text messaging administrative website. We will input what time of day to send the message based on the participant’s preference and indicate whether they should be in the smoking (“current smoker” or “quit within the past 6 months”) or nonsmoking stream. These streams have 2 different text messages where current smokers are provided with cessation information while nonsmokers are encouraged to remain smoke-free and to avoid second-hand smoke. The text message delivery will be automated, and each text message will cost $0.0075 to send.

We will be able to confirm if the text messages are delivered but not whether the text messages were opened and read. Some of the text messages contain URL links and phone numbers for resources that are accessible province-wide. The URL links are converted to a bit.ly link to make them shorter and to allow us to monitor how many times the links were accessed. After the initial sign-up, study staff involvement will only be required if the participant is readmitted to the hospital. Participants who are readmitted will have their text messages paused until discharge for all readmissions. Those participants readmitted for ACS will be restarted from the day 1 text message when discharged. Participants are instructed about the process to inform us of any readmittances at the time of consent, when we inform them of their group assignment, and in 3 text messages throughout the text messaging program. The text message is a 1-way communication; if a participant responds to the text, they will receive an automated message saying that incoming text messages are not monitored regularly. Participants receive a 1-page information sheet with instructions about the text messaging program. Participants will be able to request to stop receiving the text messages by speaking to the randomization assistant over the phone.

An advisory committee (consisting of cardiologists, a general practitioner, a community pharmacist, a cardiac nurse specialist, patient-users, a programmer, a benefits evaluation specialist from a federally funded, nonprofit digital health organization, and academic researchers) developed the messages based on 6 guiding principles. Messages had to be (1) based on clinical evidence, (2) consistent with the hospital’s current discharge instructions, (3) general enough to apply to a range of patients with the target condition, (4) within a 160-character limit to be compatible with older mobile phones, (5) at a grade 8 reading level, and (6) cocreated with patients to be acceptable. The advisory committee identified important themes to include such as the timing of the standard appointments (all ACS patients discharged from the recruitment hospital are recommended to visit their general practitioner within 2 weeks and cardiologist within 6 weeks), psychosocial needs (including depression, stress, anger, and social support), diet, physical activity, medication information, and recovery guidelines (eg, returning to work, driving, and resuming sexual activity). The intervention incorporates social marketing principles such as formative research about the target audience’s perspective and emphasis on appropriate communication channels and messages [33]. Instead of conforming to a single one of the many branded theories of behavior change, the intervention reflects a set of cross-cutting theoretical domains; the themes in the messages relate to concerns about knowledge, skills, roles and identity, beliefs about capabilities (eg, self-efficacy), beliefs about consequences, motivation, attention and decision processes (eg, cues to action such as reminders), environmental context and resources, social influences, emotion, and action plans [34].

The advisory committee drafted and revised messages based on the guiding principles, identified themes, current discharge materials, and interviews conducted with 4 discharged CVD patients (1 man and 3 women; ages 36-71 years). Revisions addressed the wording, timing, and order of the messages as well as reviewing and including any absent topics that were believed to be important based on the advisory committee’s experiences. After the advisory committee approved the messages, 2 focus groups (totaling 7 participants with coronary artery disease; 5 women and 2 men) were held with participants of a cardiac rehabilitation program to assess the appropriateness and acceptability of the messages. After further minor revisions to address the findings from the focus groups, the patient members of the clinical advisory committee pilot-tested the text messages by receiving them for 60 days.

**Table 1:** Examples of the text messages developed. All messages start with “T2P:” to indicate the source *.*

Topic	Example text message
Appointment reminders	T2P: Make an appointment to see your family doctor within 2 weeks of leaving the hospital. If you need a doctor, try the tool at: http://bit.ly/findaMD. (Day 2)
Smoking cessation	T2P: Not smoking is one of the most important things you can do for your health. For quitting resources, check out: http://bit.ly/quitnow.bc. (Day 8)
Recovery guidelines	T2P: Resuming sex: a general guide is that if you can go up a flight of stairs without symptoms, it is probably safe to restart sexual activities. (Day 14)
Psychosocial	T2P: It is common to feel sad or depressed after a heart attack or being in the hospital. If you feel this way for 2+ weeks, contact your doctor. (Day 16)
Physical activity	T2P: Have you done something physically active today? If you have questions, call the Physical Activity Line at 1-877-725-1149 or talk to your doctor. (Day 21)
Medication reminders	T2P: Bring a list of your medications to your appointment when you see your doctor. You can get copies from your pharmacist. (Day 9)

#### Usual Care

The usual care group will not receive text messages. During hospitalization, these participants will typically receive an education session from a nurse prior to discharge as well as being provided with printed educational materials. The participant is informed that they should see their general practitioner within 2 weeks, their cardiologist within 6 weeks, and are recommended to join a cardiac rehabilitation program. The follow-up appointment with the cardiologist and the referral to cardiac rehabilitation may be scheduled while the participant is in the hospital, but they generally have to schedule the appointment with their general practitioner themselves. If they wish to join any additional programs, they must seek these out or learn about them from their health care professionals.

### Study Outcomes

The primary outcome is the change in self-management between the 2 groups as measured by the heiQ. The heiQ comprises 40 questions and measures 8 domains that are indicators of effective self-management programs: positive and active engagement in life (5 questions), health-directed behavior (4 questions), skill and technique acquisition (4 questions), constructive attitudes and approaches (5 questions), self-monitoring and insight (6 questions), health service navigation (5 questions), social integration and support (5 questions), and emotional well-being (6 questions). Items are scored on a Likert scale from 1 to 4. The heiQ was developed using item response theory and structural equation modeling and the subscales have acceptable to high internal consistency (Cronbach alphas ranging from .70-.86, depending on the domain) [30]. The heiQ has been used in a broad range of patient education programs including ehealth settings and with CVD patients [35]. The heiQ will be measured at both the baseline and follow-up sessions.

Secondary outcomes are health-related quality of life, cardiac self-efficacy, medication adherence, health care resource use, hospital readmissions, and mortality. Health-related quality of life will be measured by the EQ-5D-5L, a measure of health status developed by the EuroQol Group that comprises 5 dimensions (mobility, self-care, usual activities, pain/discomfort, and anxiety/depression; 1 item per dimension) with each item having 5 levels of response [36]. The Canadian EQ-5D-5L scoring algorithm will be used to value the health state descriptions reported by study participants [37]. Cardiac self-efficacy will be measured through a modified Sullivan Cardiac Self-Efficacy scale. The original scale has 13 items and is composed of 2 factors, control symptoms and maintain function. Responses are on a 5-point Likert scale where a higher number indicates more confidence. It has high internal consistency (Cronbach alphas of .90 and .87 for the 2 scales, respectively) and good convergent and discriminant validity when compared with other distress and disability scales [38]. The modified version combines 2 questions about symptoms and adds additional questions about diet and emotional distress. Medication adherence will be measured with the 8-item medication adherence scale developed by Morisky et al [39,40,41]. This medication adherence scale has good internal consistency (Cronbach alpha = .83) and reliability when assessed in a hypertensive population [39]. It has good sensitivity (93%) and moderate specificity (53%) [39]. Health care resource use over the 60-day follow-up period (ie, visits to health care practitioners, visits to a hospital, use of phone health services, cardiac rehabilitation program participation, out-of-pocket expenses, and medication use) will be self-reported by all study participants in a structured format using a questionnaire developed by the research team [42]. Self-report resource use questionnaires provide an efficient method of collecting information in the absence of routine data sources [42,43]. Although reliance on self-reported health care resource use may be regarded as a limitation, the 60-day follow-up period in this study is significantly shorter than timeframes that have been used extensively in economic evaluations performed alongside clinical trials. Hospital readmissions will be assessed through self-report and medical records. Mortality will be assessed through medical records. The EQ-5D-5L and cardiac self-efficacy scale will be measured both at baseline and follow-up while medication adherence and health care resource use will be measured only at follow-up.

The study’s third objective is to assess the acceptability of the text messaging intervention program and to describe the feasibility of the study protocol. Acceptance of the text messaging intervention will be assessed by measuring participant satisfaction with the program using 2 5-point Likert items as well as through semistructured interviews with intervention group participants about their experiences with the text messages. During the semistructured interviews, we will explore aspects of the text messaging program that were felt to be beneficial and aspects that were considered less beneficial and could be improved. To assess feasibility, we will track recruitment rates, follow-up rates, and questionnaire completion rates. We will also evaluate the randomization process, the text message delivery system, and the data collection process by asking research staff to keep a log of barriers and challenges. Study staff will be asked to document in writing their perceptions of the acceptability and feasibility of the program along with any other feedback they wish to provide.

### Statistical Analyses

As this is a pilot study, we are not statistically powered to detect the magnitude of differences we will be looking for in the full trial. Nevertheless, we will undertake similar analyses to those we will use in the full trial and regard them as exploratory in nature. First, descriptive statistics will be used to characterize the sample. All variables will be analyzed for their distribution, and relevant transformations will be applied if distributions are nonnormal. For the primary outcome, change from baseline heiQ scores will be analyzed using multiple linear regression, using the intent to treat principle. The dependent variable will be the change from baseline heiQ scores while the independent variables will be group assignment, age, sex, and any clinically relevant baseline factors identified. Change in health-related quality of life and self-efficacy questionnaire scores and differences in medication adherence scores will be modeled in the same method as the primary outcome. Predictors of hospital readmissions and mortality will be analyzed with logistic regression while a survival analysis will be used to evaluate the time until event between the 2 groups using a Cox proportional hazards regression. Group assignment, age, and sex will be a priori covariates. Clinically relevant differences in baseline factors will be included as covariates in the models. Missing values will be addressed as per the guidelines provided with the questionnaires. SPSS (IBM Corp) will be used for statistical analysis. Statistical significance will be set at *P*<.05.

An economic evaluation will also be performed alongside the pilot trial, utilizing EQ-5D-5L and health care resource use data collected during the 60-day follow-up period. A cost-consequence analysis framework will be used as the base case economic evaluation [44,45] where resource use (and associated costs) and outcomes (eg, quality-adjusted life years [QALYs] generated from EQ-5D-5L responses) within the 2 study groups are listed, separately, in a disaggregated format. This type of evaluation makes no attempt to combine costs and effects into a single outcome measure (such as a cost-per-QALY ratio) and has particular value in aiding transparency. Canadian guidelines for the conduct and reporting of economic evaluations will be followed; publication of updated Canadian guidelines is expected in early 2017 [46].

### Assessments

#### Baseline Assessment

When possible, the baseline assessment will be done as a face-to-face session prior to hospital discharge. In situations where the baseline assessment cannot be completed prior to discharge, it will be completed by phone or through an online survey within 7 days of discharge. The assessment will consist of the heiQ, EQ-5D-5L, and Cardiac Self-Efficacy questionnaires. Participants will also be asked demographic questions (age, sex, marital status, geographic location, employment status, education, and household income) and questions about their mobile phone (frequency of use, texting frequency, need for help or support, confidence in using, type of mobile phone, and ownership). Medical history (reason for admission, comorbidities, previous cardiac events, and smoking status) will also be obtained through a combination of self-report and medical record review. Trained assessors will be blinded as randomization will not occur until after the baseline assessment.

#### Follow-up Assessment

At 60 days following randomization, all participants will be contacted to complete a follow-up assessment. The assessment will consist of the readministration of the heiQ, EQ-5D-5L, and modified Cardiac Self-Efficacy Scale. Additionally, the Morisky Medication Adherence Scale (MMAS), health care resource use and smoking status questionnaire, and the readmissions and mortality assessment will be completed. All readmission events per participant will be recorded. The health care resource use questionnaire will be administered over the phone, while the other questionnaires (heiQ, EQ-5D-5L, Cardiac Self-Efficacy Scale, and MMAS) will be sent by surface mail, along with a $20 gift card. If the participant is unable or unwilling to complete the questionnaires by mail or phone or is unresponsive to our attempts at mail or phone contact, they will have the option to complete the questionnaires via an online survey. An approved online version of the EQ-5D-5L will be used (approval from the EuroQol Group), while the other outcome measures will be adapted to an online survey format and tested for user friendliness. The method for assessment will be documented in the study database. If the participant cannot be contacted, we will attempt to recontact every 3 to 5 days by using phone, mail, and email contact information. Participants will be considered lost to follow-up if the follow-up session is not completed within 6 weeks after the 60 days following randomization.

Participants who are randomized to the Txt2Prevent group will also be invited to participate in a semistructured phone interview after completion of the 60-day follow-up. Interviews are expected to be approximately 30 minutes and will be done at a different time than the follow-up assessments in order to avoid a lengthy phone call. Interview participants will be selected to cover a range of characteristics to represent the sample of study participants (eg, male/female, rural/urban, and different age groups). Participants will be asked to share their experiences of living with and managing their condition as well as their views on the text messaging program (eg, “Can you tell me about how you have managed your heart condition over the past 2 months?” and “Has the text messaging program impacted your life over the past 2 months? If so, how?”). Questions to evaluate the text messages will ask about the clarity, tone, and frequency of the messages; the duration of the program; what topics were the least or most helpful; and what proportion of messages they read. Interviews will be recorded and transcribed verbatim and analyzed through a general inductive approach [47]. Through an iterative process, categories and themes will be created by detailed reading and line-by-line coding. Interviews will continue until theme saturation occurs [48]. NVivo (QSR International) software will be used for qualitative analysis. The findings from the interviews will be valuable in providing the context for the quantitative findings and for assessing the acceptability of the intervention.

## Results

Ethics and institutional approval have been obtained from the Providence Health Care Research Ethics Board and Simon Fraser University’s Office of Research Ethics. Study staff have been hired and trained. Recruitment started in June 2015 and was completed by December 2016, with data collection being completed by January 2017.

## Discussion

### Summary

This study aims to evaluate whether a text messaging program can help support patients with ACS after their discharge from hospital. A randomized controlled pilot trial with semistructured interviews will be used to determine preliminary efficacy, feasibility, and acceptability. Although previous studies have looked at text messaging in CVD patients, no known studies have evaluated the use of text messages among patients with ACS during the hospital-to-community transition period, which is a high-risk time for readmission [6].

### Study Considerations

The Txt2Prevent study is an exploratory pilot study to assess preliminary efficacy, acceptability, and feasibility of a text messaging program to improve self-care and management in ACS patients after discharge, which leads to several study considerations. First, due to the nature of the intervention, the participants are not blinded. Additionally, it is difficult to create a suitable attention control, so the intervention group is being compared to usual care. Second, we are unable to objectively determine adherence or even if participants read the messages; however, we will ask participants about their experiences in semistructured interviews. Third, several of the outcomes are self-reported, which may introduce bias; however, the study will use common and validated measures.

### Conclusion

The Txt2Prevent study is a novel project to determine if text messaging can support ACS patients in the critical period immediately after discharge. We intend to use the results of the study to inform a larger clinical trial. If effective, the Txt2Prevent program has the potential to be translated into practice and be scaled up and implemented in clinical settings. Implementing the program on a larger scale is likely to be feasible because the program requires limited human resources and text messages are low cost. The study will contribute to our understanding of mHealth in health services research and will inform future studies on the use of text messaging to support ACS patients as they transition from hospital to home.

## Abbreviations

ACS: acute coronary syndrome
AMI: acute myocardial infarction
CIHR: Canadian Institutes of Health Research
CONSORT: Consolidated Standards of Reporting Trials
CVD: cardiovascular disease
heiQ: Health Education Impact Questionnaire
MMAS: Morisky Medication Adherence Scale
MSFHR: Michael Smith Foundation for Health Research
QALY: quality-adjusted life year

## Appendix

Multimedia Appendix 1 CIHR Peer Review.

Multimedia Appendix 2 CONSORT EHEALTH Check List.
