# Supplementary material for: The Use of Text Messaging to Improve the Hospital-to-Community Transition in Acute Coronary Syndrome Patients (Txt2Prevent): Intervention Development and Pilot Randomized Controlled Trial Protocol
Source: JMIR Res Protoc. 2017 May 23;6(5):e91. doi: 10.2196/resprot.6968 (PMC5461423; doi:10.2196/resprot.6968)
Supplement: Multimedia Appendix 1 [file resprot_v6i5e91_app1.pdf]

February/Février 21, 2014

DC0190GP

Canadian Institutes of Health Research / Instituts de recherche en santé du Canada

Notice of Decision / Avis de décision

Application Number/Numéro de la demande: 316822

Committee Code/Code du comité: EHI

Applicants/Candidats: Dr. Scott A LEAR

With/Avec: Prof. A. HAGAN-JOHNSON, Dr. M. MACKAY, Mr. B. SAKAKIBARA, Dr. M. TOMA, Dr. H. VAN SPALL, Dr. D. WHITEHURST

Institution paid/ Établissement payé: Simon Fraser University (Burnaby, B.C.)

Title/Titre: The use of text messaging to improve the hospital-community transition and prevent readmission in patients with cardiovascular disease (Txt2Prevent)

Primary Inst./ Inst. principal: Health Services and Policy Research

Other Related Inst./ Autres inst. connexes: Circulatory and Respiratory Health; Population and Public Health

**Competition Outcome/Résultats du concours:**	Catalyst Grant: eHealth Innovations<br>October/Octobre 15, 2013
**Number in competition/Nbre de demandes dans le concours:**	88
**Number approved/Nbre de demandes approuvées:**	12

**Decision on your application/ Décision sur votre demande:**	Approved
**Average annual amount/ Montant annuel moyen:**	$78,192
**Equipment amount/ Montant pour les appareils:**	$1,383

Peer Review Committee Recommendation, for your information and use/ Recommandation du comité d'examen par les pairs, pour fins d'information et d'utilisation:

**Committee/Comité:**	e-Health Innovations
**Application rank within the competition/ Rang de la demande dans ce concours:**	13
**Percent Rank Within the Competition/ Rang en pourcentage au sein du concours:**	14.77%
**Rating/ Cote:**	3.84
**Recommended average annual amount/ Montant annuel moyen recommandé:**	$78,192
**Recommended equipment amount/ Montant recommandé pour les appareils:**	$1,383

*** Applications receiving a score of less than 3.5 on any evaluation criteria will not be considered for Funding. / Les demandes qui ont reçu une note inférieure à 3.5 pour n'importe quel des critères d'évaluation ne sont pas admissibles.

This document is for information only. Official payment is stated on the CIHR Authorization for Funding.
Document à titre d'information seulement. Le paiement officiel est indiqué sur l'autorisation de financement des IRSC.

Review Type/Type d'évaluation: Committee Member 1/Membre de comité 1
Name of Applicant/Nom du chercheur: LEAR, Scott A
Application No./Numéro de demande: 316822
Agency/Agence: CIHR/IRSC
Competition/Concours: 2013-10-15 Catalyst Grant: e-Health Innovations: Supporting More Efficient Population and Individualized Healthcare/Subvention catalyseur : Innovations en cybersanté : soutenir l'amélioration des soins de santé axés sur les individus et les populations
Committee/Comité: e-Health Innovations/Innovations en cybersanté
Title/Titre: The use of text messaging to improve the hospital-community transition and prevent readmission in patients with cardiovascular disease (Txt2Prevent)

Assessment/Évaluation:

The use of text messaging to improve the hospital-community transition and prevent readmission in patients with cardiovascular disease

PI: Lear Scott, Faculty of Science, Simon Fraser University (4 hrs/week)
Co-Applicants:

- Hagan-Johnson Alyson, Knowledge User, Unknown (1 hr /week)
- MacKay Martha Helen, Co-Applicant, School of Nursing, U of BC (1 hr /week)
- Sakakibara Brodie Masaru, Co-Applicant, Faculty of Sciences, Simon Fraser U (2 hrs/week)
- Toma Mustafa, Co-Applicant, Cardiology, Uof BC (1 hr/week)
- Van Spall Harriette, Co-Applicant, Cardiology, McMaster U (1 hr/week)
- Whitehurst David George Thomas, Co-Applicant, Faculty of Health Sciences, McMaster University (2 hrs/week)

<u>Synopsis of the Proposal</u>

- Purpose: To develop and pilot-test a program that send text messages to patients within the first 60 days after hospital discharge. The test messages will be sent at regular intervals and will prompt the patient on follow-up care, medication use and healthy lifestyle behaviors.
- Single-blinded Randomized trial
    - Intervention group: a hospital-community transitioning program using health text-messaging
    - Control group: usual care – participants will receive non-health text messaging in addition to standard transitional care.
- Assess patient self-management, medication use, quality of life, and satisfaction with the program, as well as costs to the health care and patient.
- Txt2Prevent will consist of a password-protected, web-based registration interface that will allow the research coordinator to register and manage study participants.
- Txt2Prevent will be pre-programmed to send automated, timely and relevant reminders, tips and knowledge over a 60-day period via text-messages to improve: adherence with medications and follow-up care, and support patient self-management and promote healthy lifestyle behaviors, key areas

Review Type/Type d'évaluation: Committee Member 1/Membre de comité 1
Name of Applicant/Nom du chercheur: LEAR, Scott A
Application No./Numéro de demande: 316822
Agency/Agence: CIHR/IRSC
Competition/Concours: 2013-10-15 Catalyst Grant: e-Health Innovations: Supporting More Efficient Population and Individualized Healthcare/Subvention catalyseur : Innovations en cybersanté : soutenir l'amélioration des soins de santé axés sur les individus et les populations
Committee/Comité: e-Health Innovations/Innovations en cybersanté
Title/Titre: The use of text messaging to improve the hospital-community transition and prevent readmission in patients with cardiovascular disease (Txt2Prevent)

Assessment/Évaluation:

that have been identified in the literature to cause hospital readmissions in CVD patients.

- Txt2Prevent will be developed in the first 3 months of the project under guidance of the clinical advisory committee that will consist of patients, allied health professionals, cardiologists, IT specialists and members of the research team.
- Hypothesis:
  - Primary: relative to usual care, patients with CVD who participate in Txt2Prevent will have lower combined hospital readmission and mortality rates over 60 days post-discharge
  - Secondary: participation in Txt2Prevent will improve self-efficacy in self-management, medication adherence, health-related quality of life, attitudes, experiences, and satisfaction with care while resulting in lower healthcare and patient costs.
- Primary outcome: difference in combined 60-day cardiac hospital readmissions and mortality between the 2 groups based on self-report and confirmed through patient medical records.
- Secondary outcomes: 1) self-efficacy using the 12-item Cardiac Self-efficacy scale, 2) medication adherence measured using the 8-itme self-report Medication Adherence Scale, 3) health-related quality of life measured using the EQ-5D-5L, 4) patient attitudes, experience, and satisfaction using semi-structured interviews, 5) healthcare and patient costs.
-
- <u>Assessment of the Proposal</u>

- Industry partner: CISCO Systems – letter of support
- Recruitment procedure is described.
- "The final numbers of interviews will be driven by the prospective discovery of factors (or concepts) that may affect participants satisfaction with care provided by Txt2Prevent." Unclear.
- Examples of non-healthy text messages?
- No psychometric characteristics of the scale or measurements used are provided.
- Quantitative data analysis well described however the qualitative data analysis is not specified. Will interviews be audiotaped, transcribed, content analyzed?
- Nothing is specified how the text message will be developed and how from a technical standpoint it will be evaluated before conducting the pilot study.
- A health economist is part of the team – Dr. David Whitehurst.
- Well-defined study timeline.
- A sample size of 68 will be used for the quantitative study. In the budget justification the researchers

**Review Type/Type d'évaluation:**	Committee Member 1/Membre de comité 1
**Name of Applicant/Nom du chercheur:**	LEAR, Scott A
**Application No./Numéro de demande:**	316822
**Agency/Agence:**	CIHR/IRSC
**Competition/Concours:**	2013-10-15 Catalyst Grant: e-Health Innovations: Supporting More Efficient Population and Individualized Healthcare/Subvention catalyseur : Innovations en cybersanté : soutenir l'amélioration des soins de santé axés sur les individus et les populations
**Committee/Comité:**	e-Health Innovations/Innovations en cybersanté
**Title/Titre:**	The use of text messaging to improve the hospital-community transition and prevent readmission in patients with cardiovascular disease (Txt2Prevent)

Assessment/Évaluation:

estimate 20 interviews. Nothing is mentioned in the proposal and how will they select those potential 20 participants from the intervention group for the interview.

- The majority of the team members will spend about 1-2 hrs/week on this project. May have implications with regard to the feasibility of the study.

Budget

- Request CIHR 84,768$ - OK
- 1 Research coordinator, 1 Research Assistant (randomization), 1 RA (cost analysis), 1 Graduate student
- Statistical consultant? $$ for other personnel?
- No In-kind contributions

-

Review Type/Type d'évaluation: Committee Member 2/Membre de comité 2
Name of Applicant/Nom du chercheur: LEAR, Scott A
Application No./Numéro de demande: 316822
Agency/Agence: CIHR/IRSC
Competition/Concours: 2013-10-15 Catalyst Grant: e-Health Innovations: Supporting More Efficient Population and Individualized Healthcare/Subvention catalyseur : Innovations en cybersanté : soutenir l'amélioration des soins de santé axés sur les individus et les populations
Committee/Comité: e-Health Innovations/Innovations en cybersanté
Title/Titre: The use of text messaging to improve the hospital-community transition and prevent readmission in patients with cardiovascular disease (Txt2Prevent)

Assessment/Évaluation:

LEAR - The use of text messaging to improve the hospital-community transition and prevent readmission in patients with cardiovascular disease (Txt2Prevent)

A brief synopsis of the proposal

A proposal to evaluate an SMS-based approach to discharge followup.

Strengths

There are many strengths to this proposal. Firstly it is well supported and includes a very qualified multidisciplinary team well suited to this task. It fits well with the grant objectives (with one exception below), has the real potential for impact and addressing an important need. It is clear and well described regarding its intent and deliverables. The authors should be commended

Limitations

I only observed two important limitations with the proposal. First and foremost, nowhere in the proposal is described commercialization and innovation in e-health opportunities. The only thing I can see is that will further strengthen a non-commercial project (BCATPR)

The second limitation worth mentioning is nowhere is it described how the intervention will be delivered. What infrastructure will be used etc. I would imagine the need to deliver large amounts of SMS is an opportunity for some commercial collaboration.

1. **Comments on the budget requested and a formal recommendation**, including clear and detailed reasons for any recommended budget cuts.

2. **If necessary, comments on issues the reviewer feels should be flagged** (Section 6.2.5). These concerns should not influence the rating or budget recommendations, unless they bear on the scientific merit of the application.

Review Type/Type d'évaluation: SO Notes /Notes de l'agent scientifique
Name of Applicant/Nom du chercheur: LEAR, Scott A
Application No./Numéro de demande: 316822
Agency/Agence: CIHR/IRSC
Competition/Concours: 2013-10-15 Catalyst Grant: e-Health Innovations: Supporting More Efficient Population and Individualized Healthcare/Subvention catalyseur : Innovations en cybersanté : soutenir l'amélioration des soins de santé axés sur les individus et les populations
Committee/Comité: e-Health Innovations/Innovations en cybersanté
Title/Titre: The use of text messaging to improve the hospital-community transition and prevent readmission in patients with cardiovascular disease (Txt2Prevent)

Assessment/Évaluation:

LEAR

This is a well written proposal from a highly qualified team. More justification about the potential effectiveness of the intervention in decreasing readmission for this patient group would strengthen the application. A description of "non-healthy" text messages is needed. There were limited details on the delivery and timing of the intervention and the analysis of qualitative data. The committee was concerned about the limited time that team members will dedicate to the project.
